# Right-Sided Versus Left-Sided Colon Cancer—A 5-Year Single-Center Observational Study

**DOI:** 10.3390/cancers17030537

**Published:** 2025-02-05

**Authors:** Julia Szostek, Michał Serafin, Magdalena Mąka, Beata Jabłońska, Sławomir Mrowiec

**Affiliations:** 1 Student Scientific Society, Department of Digestive Tract Surgery, Faculty of Medical Sciences in Katowice, Medical University of Silesia, 14 Medyków Street, 40-752 Katowice, Poland; s81394@365.sum.edu.pl (J.S.); michal.j.serafin@gmail.com (M.S.); s81151@365.sum.edu.pl (M.M.); 2 Department of Digestive Tract Surgery, Faculty of Medical Sciences in Katowice, Medical University of Silesia, 14 Medyków Street, 40-752 Katowice, Poland; mrowasm@poczta.onet.pl

**Keywords:** right colon cancer, left colon cancer, surgical treatment, overall survival

## Abstract

**Background:** Global colorectal cancer (CRC) incidence is significant, constituting 15% of all cancer cases with 1.4 million new diagnoses annually. Recent research suggests categorizing CRC into three clinical groups: right colon cancer (RCC), left colon cancer (LCC), and rectal cancer, each with distinct embryological and molecular characteristics. **Methods**: A retrospective analysis of 189 patients (103 men, 86 women) undergoing surgery for RCC and LCC from January 2018 to December 2023 was performed. **Results**: LCC was a more common localization (98, 51.85%) than RCC (91, 48.15%). Patients with RCC were older than patients with LCC (70 (36–92, IQR 11) vs. 68 (38–84, IQR 12.5) years; *p* = 0.02). The duration of surgical procedure was comparable in both groups (225 (120–420, IQR 80) vs. 210 (105–505, IQR 85) minutes; *p* = 0.16). Complications occurred in 16 (17.58%) patients with RCC and in 15 (15.31%) patients with LCC (*p* = 0.72). One-year overall survival was 92.76% (SE 2.16%) (91.57% (SE 3.43%) in the RCC group and 93.99% (SE 2.61%) in the LCC group; *p* = 0.79). **Conclusions:** Colon cancer incidence is increasing globally due to economic and lifestyle factors. Our study reflects this trend, noting a rise in cases from 2018 to 2023. Despite several differences, overall survival rates do not significantly differ between RCC and LCC patients. Understanding clinical disparities is crucial for optimizing patient outcomes.

## Simple Summary

Colon cancer is a significant global health concern, and understanding how tumors on the right and left sides of the colon differ is crucial for improving treatment strategies. This study analyzed five years of patient data, examining differences in surgical treatments, complication rates, and survival outcomes for 189 cases of right- and left-sided colon cancer. Right-sided tumors were found more often in older patients and were more frequently mucinous in type, while left-sided tumors were predominantly adenocarcinomas. Despite these distinctions, surgical complication rates, treatment approaches, and overall survival outcomes were comparable between both groups. The number of lymph nodes removed was notably higher in right-sided cases, likely reflecting more extensive surgical procedures. These findings demonstrate that while tumor location influences clinical and pathological characteristics, survival rates remain similar. The study underscores the need for continued exploration of tailored treatment approaches to enhance colon cancer management and optimize long-term patient outcomes.

## 1. Introduction

Globally, colorectal cancer (CRC) constitutes 15% of all cancers. Approximately 1.4 million new cases of colorectal cancer are reported annually. Its incidence is especially high in many high-income countries [1,2]. In 2021, colorectal cancer was the third most common diagnosed cancer in Poland, while in Italy it was the second most malignant tumor with approximately 13% of all cases [3,4]. Rabbani et al., have observed that CRC is beginning to affect the younger population, posing a growing problem for public health [5].

The latest studies suggest a possibility that CRC should be divided into three groups from a clinical management perspective: right colon cancer (RCC), left colon cancer (LCC), and rectal cancer [6]. There are many differences that support this division. Firstly, RCC, LCC, and rectal cancer develop from two different embryological guts—the RCC from midgut, and it is supplied by the superior mesenteric artery, while LCC and rectal cancer develop from the hindgut, and they are being supplied by the inferior mesenteric artery. From the histological and molecular point of view, RCCs are more often mucinous or signet ring cell tumors with worse differentiation than LCCs [7,8]. In addition, microsatellite instability, BRAF mutations, and RAS mutations have higher incidence in RCC compared to LCC [9,10]. The study conducted by Brouwer et al. showed that radiological staging, neoadjuvant chemotherapy, as well as a surgical techniques can be improved by separating the rectal cancer from RCC and LCC, therefore, improving the patient’s outcome [2].

Surgical and oncological outcomes have been reported to be worse in RCC than in LCC [7,11]. West et al. suggest that in the surgical resection of LCC, it is possible to maintain greater radicality and therapeutic success of the procedure, which is subsequently associated with reduced 5-year mortality [12]. On the other hand, Weiss et al. have reported no significant difference in 5-year mortality between RCC and LCC [13]. Recent studies seek for intraoperative differences between RCC and LCC, which may directly translate into increased overall survival. The study conducted by Bernhoff et al. showed that in RCC, performance of complete mesocolic excision (CME) over standard right hemicolectomy may increase the recurrence-free survival [14]. Similar conclusions can be found in the study of Bertelsen et al. [15]. Furthermore, Lee et al. demonstrate that patients with RCC with at least 12 lymph nodes harvested during the surgical procedure have lower predicted survival compared with patients with LCC with at least 12 lymph nodes harvested. Nonetheless, the highest predicted survival was observed among patients with RCC and >21 lymph nodes harvested [16]. Nevertheless, the underlying mechanisms between cancer localization and overall survival remains unclear [17].

The primary objective of this study is to conduct a clinical, pathological characterization as well as a comparison between right colon cancer (RCC) and left colon cancer (LCC). Comparing the perioperative, short-term, and long-term outcomes of surgical interventions between RCC and LCC. The data for this comparative analysis are sourced from the Department of Digestive Tract Surgery at the Medical University of Silesia in Katowice, Poland.

## 2. Materials and Methods

### 2.1. Study Design and Population

Retrospective analysis included all patients who underwent surgery for colon cancer from January 2018 to December 2023 in the Department of Digestive Tract Surgery, Medical University of Silesia in Katowice, Poland. The electronic medical records for each patient individually were reviewed.

Inclusion criteria were primary surgical colon resection in elective or emergency mode and cancer confirmed by histopathological finding. Exclusion criteria were as follows: rectal/rectosigmoid junction cancer, recurrent colon cancer, and lack of postoperative follow-up.

The study group consisted of 189 adult patients (103; 54.50% males and 86; 45.50% females), aged 36–92 (69, IQR 11).

### 2.2. Inclusion Criteria to Surgical Treatment

All patients underwent evaluation by a multidisciplinary team consisting of surgeons, radiologists, and oncologists, who collectively determined the appropriate qualification for a specific type of treatment based on computed tomography (CT) findings. Moreover, 173 (91.53%) patients underwent endoscopic biopsy with subsequent histopathological diagnosis of colon cancer prior to surgical intervention.

The nationwide screening program in Poland, implemented in 2000, offers colorectal cancer screening for patients aged above 50 years. However, individuals from the age of 45 are eligible for the screening if they have a family history of colorectal cancer or other risk factors, such as hereditary conditions like Lynch syndrome or familial adenomatous polyposis (FAP). The screening includes colonoscopy every 10 years, though participation is voluntary.

The cohort was divided into two subgroups according to the tumor localization as right colon cancer (RCC) (ceacum, ascending colon and proximal two thirds of transverse colon) and left colon cancer (LCC) (distal third of transverse colon, descending colon, sigmoid colon).

### 2.3. Analyzed Data

The study analyzed various parameters, including patients’ general characteristics (such as age, gender, and body mass index (BMI)), clinical symptoms, American Society of Anesthesiologists (ASA) score, type and duration of surgery, surgical margin status (resection R0/R1/R2), incidence of postoperative complications, reoperations, mortality, and duration of hospitalization. Additionally, parameters such as rehospitalizations, primary tumor localization and diameter, selected pathological features (including lymphovascular and perineural invasion, histopathological grading), lymph node and distant metastases, as well as follow-up data were also included in the analysis.

The patients’ general characteristics as well as incidence of postoperative complications and reoperations were obtained from the patient’s medical history.

The localization of the tumor as well as the duration and type of the procedure were collected from the description of the patient’s surgery.

Histopathological data were collected from our institution’s internal electronic pathology system.

Follow-up data were obtained from the patient’s medical history from the surgical clinic and/or from the department.

### 2.4. Definitions

Family history of colon cancer was defined as a confirmed diagnosis of colon cancer in the patient’s first-degree or second-degree relatives, including grandparents, parents, siblings, or children.

The resection margin status was categorized as follows: R0 indicated no cancer cells detected in either microscopic or macroscopic examination of the resection margin; R1 referred to cancer cells identified exclusively in the microscopic evaluation of the resection margin; and R2 denoted the presence of cancer cells in both microscopic and macroscopic assessments of the resection margin.

Primary anastomosis was defined as the surgical creation of an intestinal anastomosis during the initial procedure following the resection of a segment of the colon containing the tumor.

Emergency admission was characterized as hospital admission necessitated by acute symptoms or complications requiring immediate treatment intervention.

Complications were defined as any adverse event occurring within 30 days postoperatively, including but not limited to surgical site infections, anastomotic leakage, intestinal obstruction, and intra-abdominal abscesses. These definitions were applied consistently throughout the study to standardize data collection and analysis.

Overall survival (OS) was measured from the date of surgical procedure to either the date of death or the date of the last contact.

### 2.5. Statistical Analysis

Statistical analyses were conducted utilizing the Statistica^®^ software, version 13.3 (StatSoft, Tulsa, OK, USA). Qualitative variables were presented as absolute values and percentages, while quantitative variables were described using ranges, means and standard deviations, or medians with interquartile ranges. The Shapiro–Wilk test was applied to assess the statistical distribution among the patients analyzed. Univariate logistic regression analysis was used to identify predictive factors for postoperative complications. Subsequently, multivariate logistic regression analysis was conducted with the variables found significant in the univariate analysis to determine the independent predictors of postoperative complications following surgical treatment of colon cancer. Comparisons between groups were performed for two localization categories (RCC vs. LCC), analyzing general patient characteristics, surgical characteristics, tumor characteristics, and follow-up. The chi-square test, Fisher’s exact test, or Mann–Whitney U test were utilized for these comparisons. Survival analysis was performed using the Kaplan–Meier estimator, and prognostic factors were analyzed with the Cox proportional hazards regression model. A *p*-value of less than 0.05 was considered statistically significant.

## 3. Results

### 3.1. Patient’s Demographics and General Characteristics

**Figure 1:**
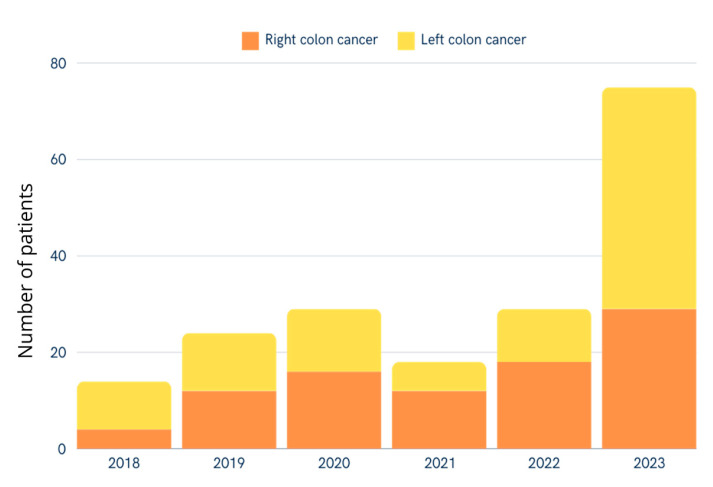
Number of patients undergoing colectomy for right-sided or left-sided colon cancer for from January 2018 and December 2023.

The number of patients treated for colon cancer has increased over the years from 14 (7.41%) in 2018 to 75 (39.68%) in 2023. The decline in the number of patients in 2021 may be linked to the COVID-19 pandemic, which has reduced access to healthcare and consequently reduced the number of patients treated for colon cancer. The rise in the number of patients in 2023 can be attributed not only to the lingering effects of the COVID-19 pandemic but also to the increased access to healthcare services after the pandemic, following a period when diagnostic and treatment services were significantly limited (Figure 1).

Patients with RCC were older compared to the LCC group (70 (IQR 11) vs. 68 (IQR 12.5) years; *p* = 0.02).

Anemia was more frequently reported in the RCC group compared to the LCC group (20 (21.74%) vs. 6 (6.19%); *p* = 0.002). In total, seven (15.56%) RCC patients and five (18.52%) LCC patients had low albumin plasma concentrations.

None of the patients in the RCC and LCC group underwent preoperative chemo- or radiotherapy (Table 1).

### 3.2. Surgical Characteristics and Outcome

Most patients were admitted in elective mode (171; 90.48%). Most patients (91; 48.15%) were assessed in the III group of ASA (Table 2).

Radical tumor resection was performed in 176 (93.12%) patients, 88 (96.70%) patients with RCC and 88 (89.80%) patients with LCC; *p* = 0.08. Palliative resection was performed in 13 (6.88%) patients (3 (3.30%) in the RCC group and 10 (10.20%) in the LCC group; *p* = 0.08). Due to the presence of distant metastasis, radical tumor resection was not possible. The most common type of surgical approach was laparotomy (103; 54.50%). In the RCC group, the most common type of the surgery was right hemicolectomy (71; 78.02%), while sigmoidectomy was the most common type of surgical treatment in the LCC group (69; 70.41%). In 187 (98.94%) patients, surgical margin status was R0. In two (1.06%) LCC patients, due to the malignant infiltration of surrounding structures (uterus (*n* = 1) and the iliac artery (*n* = 1)), the margin status was R2. Colectomy with primary anastomosis was performed in 153 (80.95%) patients (79 (86.81%) in the RCC group and 74 (75.51%) in the LCC group; *p* = 0.05). The most common anastomosis in LCC was side-to-side ileo-transversostomy (60; 65.94%) followed by end-to-end colorectal anastomosis (descending colon to rectum) (45; 48.98%). In addition, in five (2.65%) patients, the synchronous liver metastases were simultaneously resected (two (2.20%) in the RCC group and three (3.06%) in the LCC group; *p* = 1). Four (2.12%) patients underwent anatomical liver resection (three (1.59%) segmental resection of fourth liver segment and one (0.53%) resection of the seventh liver segment), and one (0.53%) patient underwent non-anatomical partial resection of the right liver lobe.

A total of 31 (16.40%) complications were observed. Thirty day mortality was 2.12%. Detailed information regarding all complications are presented in Table 2.

In univariate logistic regression analysis, occurrence of postoperative complications was associated with admission mode (emergency mode, *p* < 0.001, odds ratio (OR) = 6.77, 95% confidence interval (CI) = 2.41–19.03) and with intent of surgery (palliative resection, *p* = 0.046, OR = 3.61, 95% CI = 1.09–11.97). The multivariate logistic regression analysis showed that only the admission mode (emergency mode, *p* < 0.001, OR = 6.24, 95% CI = 2.18–17.86) was an independent predictive factor for postoperative complications (Table 3).

In total, four (4.30%) reoperations were performed in the RCC group, while reoperation was needed in six (6.12%) LCC. (Table 4).

### 3.3. Tumor Characteristics

The most common tumor localization in the RCC group was ascending colon (32; 35.16%), while sigmoid colon was the most common localization in the LCC group (75; 76.53%). Median tumor size was 38 (2–450, IQR 27).

In 179 (94.71%) patients, the cancer histological type was adenocarcinoma (82 (90.11%) in the RCC group and 97 (98.98%) in the LCC group; *p* = 0.008). The mucinous carcinoma was more often observed in patients with RCC compared to the patients with LCC (nine (9.89%) vs. one (1.02%); *p* = 0.008). Most tumors were classified as histological grade 2 (143; 75.66%). In pathological staging, 81 (42.86%) tumors were classified as T3. lymph node metastasis (N1) was found in 39 (20.63%) patients, while distant metastasis (M1) was found in 18 (9.52%) patients. Most tumors (66; 34.92%) were diagnosed as stage I. The most common localization of distant metastasis was liver (15; 7.94%). In RCC patients, the number of isolated lymph nodes was higher than in patients with LCC (17 (1–45, IQR 12) vs. 13 (1–57, IQR 10); *p* = 0.002). This fact can be associated with the extension of surgical resection. In RCC, the most common type of surgery was right hemicolectomy (71; 78.02%), in which the extent of resection is greater than in sigmoidectomy (the most common type of surgery in LCC (69; 70.41%) (Table 5).

### 3.4. Follow-Up

Median follow-up time was 11 (0.5–63.50, IQR 23.50) months.

In total, 43 (22.75%) patients underwent adjuvant chemotherapy (12 (13.19%) patients in the RCC group and 31 (31.63%) patients in the LCC group; *p* = 0.003). The most common type of chemotherapy was FOLFOX4 (13; 6.88%) and FOLFIRI (13; 6.88%). Patients with LCC more often underwent FOLFOX4 chemotherapy compared to the patients with RCC (11 (11.22%) vs. 2 (2.20%); *p* = 0.02). Additionally, three (1.59%) patients underwent radiotherapy, and all radiotherapy was performed in patients with LCC (3; 3.06%). The radiotherapy was performed due to the R2 surgical margin (2; 1.06%) and R0 surgical margin with less than 1 mm (1; 0.53%)

**Figure 2:**
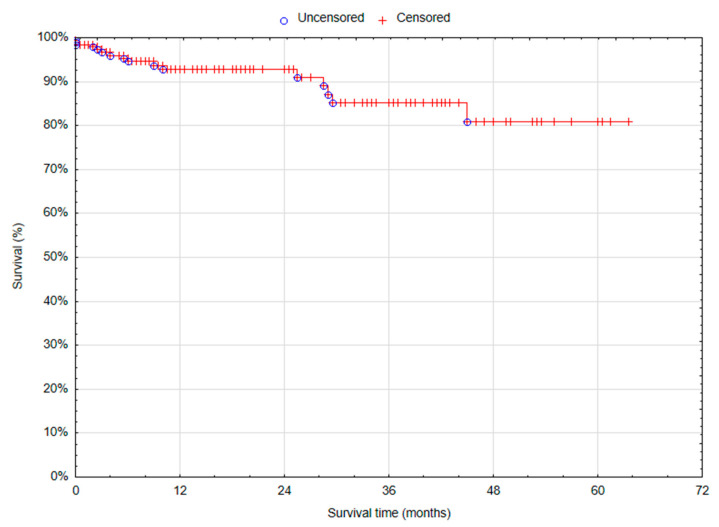
Overall survival rate of the series.

**Figure 3:**
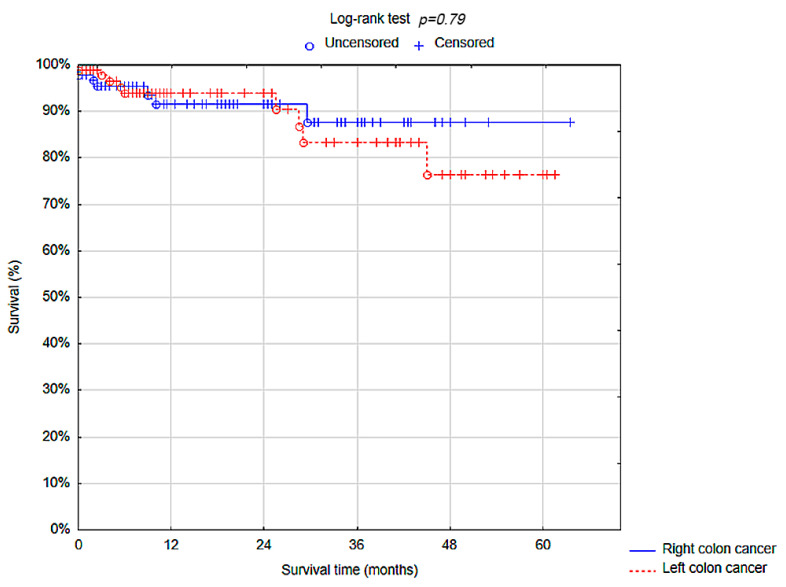
Overall survival rate—right colon cancer vs. left colon cancer.

The cancer recurrence was observed in two (1.06%) patients; all recurrence was noted in patients with LCC (2; 2.04%). One-year overall survival (OS) in total was 92.76% (standard error (SE) 2.16%) (Figure 2). One-year OS was comparable between RCC and LCC groups (91.57% (SE 3.43%) vs. 93.99% (SE 2.61%); *p* = 0.79). The estimated 5-year overall survival was 85.25% (SE 4.11%) (87.58% (SE 5.10%) in the RCC group and 76.33 (SE 8.79%) in the LCC group; *p* = 0.79 (Table 6) (Figure 3).

**Figure 4:**
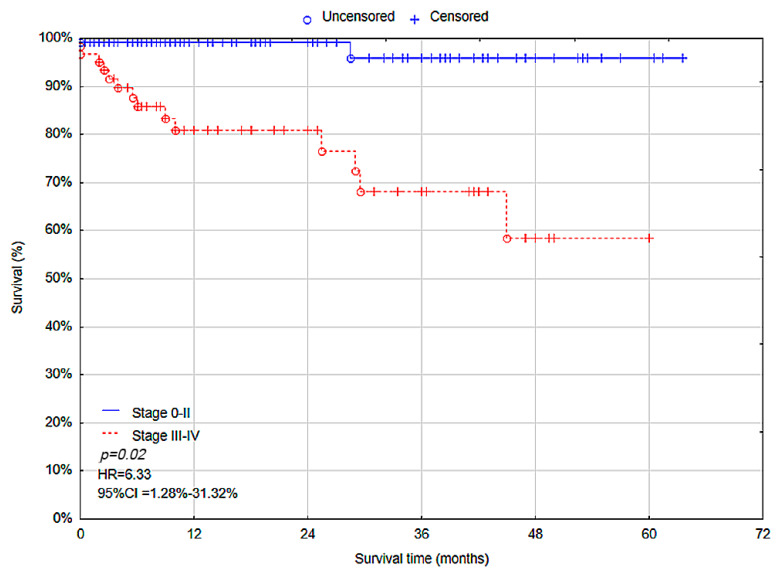
Overall survival rate—Stage 0–II vs. Stage III–IV.

**Figure 5:**
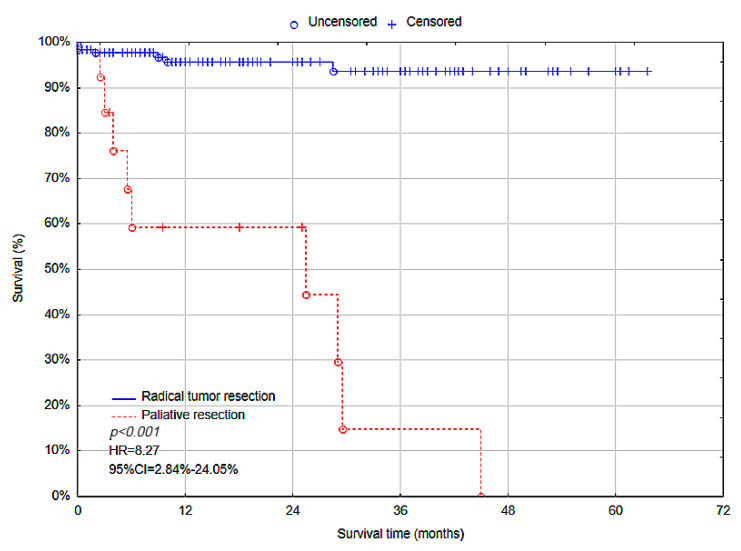
Overall survival rate—Radical tumor resection vs. Palliative tumor resection.

In univariate Cox proportional hazards analysis, overall survival was associated with tumor stage (stage III–IV, *p* < 0.001, hazard ratio (HR) = 13.37, 95% CI = 3.04–58.89) and scope of surgery (palliative resection, *p* < 0.001, HR = 18.44, 95% CI = 6.85–49.66). The multivariate Cox proportional hazards analysis showed that tumor stage (stage III–IV, *p* = 0.02, HR = 6.33, 95% CI = 1.28–31.32) (Figure 4) and scope of surgery (palliative resection, *p* < 0.001, HR = 8.27, 95% CI = 2.84–24.05) (Figure 5) were independent predictive factors for overall survival. Therefore, AJCC stage III–IV and palliative resection were associated with higher risk of death among patients (Table 7).

## 4. Discussion

Our study revealed an increasing incidence of colon cancer cases over the analyzed period, rising from 14 cases (7.41%) in 2018 to 75 cases (39.68%) in 2023, with a median age of diagnosis at 69 years. Patients with right-sided colon cancer (RCC) were older compared to those with left-sided colon cancer (LCC) (70 vs. 68 years, *p* = 0.02). Clinical symptoms were present in 56.61% of patients, with anemia significantly more frequent in RCC (21.74%) compared to LCC (6.19%, *p* = 0.002). Postoperative complications occurred in 16.40% of patients, with no significant differences between RCC and LCC groups (*p* = 0.72). Resection margins were predominantly R0, achieved in 98.94% of cases, and RCC procedures resulted in a higher median number of lymph nodes removed compared to LCC (17 vs. 13, *p* = 0.002). There were no significant differences in overall survival (OS) between RCC and LCC, with a one-year OS of 92.76% for the entire cohort. AJCC stage III–IV and emergency admission emerged as independent predictors of poorer outcomes.

In Western countries (e.g., Canada, Sweden, and the United Kingdom), the trend of increasing incidence of colon cancer can be observed [18]. In Poland, based on the Polish National Cancer Registry, a similar trend can be found [3]. Our study aligns with the data from Polish National Cancer Registry and Western trends with increasing incidence of colon cancer from 14 (7.41%) in 2018 to 75 (39.68%) in 2023. This trend may be related to the economic development of Poland and the adaptation of Western lifestyles, which increases the number of risk factors such as obesity and dietary habits. In addition, the Poland nationwide screening program implemented in 2000 can explain the increasing numbers of diagnosed colon cancers [19,20].

Colon cancer is strongly associated with age, with a median patient age ranging from 67 to 71.3 years according to recent studies [21,22]. In our study, the median age was 69 years, with RCC patients being older than LCC patients (70 [IQR 11] vs. 68 [IQR 12.5] years; *p* = 0.02). Similar findings are reported in the literature, where RCC occurs in older patients (e.g., 73.2 years for RCC vs. 70.3 years for LCC) [21,22]. Saltzstein et al. suggest a left-to-right shift in colon cancer prevalence after age 70, likely due to altered transit time or colonic content concentration in the right colon, leading to prolonged carcinogen exposure and increased carcinogenesis. However, the underlying mechanisms remain unclear [23,24].

Symptoms of colon cancer vary depending on tumor localization. The overall symptom prevalence in the literature ranges from 61.70% to 95.1% and shows no significant differences between RCC and LCC [25,26]. In our study, the prevalence was lower at 56.61%, likely due to 173 (95.05%) patients being diagnosed through endoscopic biopsy before surgery, and 82 (43.39%) asymptomatic cases were identified via screening colonoscopy. Common symptoms include abdominal pain (27.69–52%), weight loss (4.62–39%), rectal bleeding (12.30–58%), and anemia (17.25–57%) [25,26,27]. Abdominal pain and weight loss are more frequent in RCC (33.3–66.7% and 32.2%, respectively) compared to LCC (33.33–40.8% and 20.6%), while rectal bleeding is more common in LCC (23.07–61.9%) than RCC (16.67–25.8%) [25,26,28]. In our cohort, abdominal pain (31.75%), weight loss (21.69%), hematochezia (14.81%), and anemia (13.76%) were observed, with anemia being significantly more frequent in RCC compared to LCC (21.74% vs. 6.19%; *p* = 0.002). The high proportion of elective admissions (171; 90.58%) and asymptomatic screening likely influenced these findings.

In the literature, a notable trend suggests that patients with LCC are more often admitted in emergency mode compared to those with RCC. Mik et al. reported emergency admission rates of 17% for LCC and 8.5% for RCC [29]. However, other studies, such as Yang et al., did not find significant differences, with 17.9% of LCC and 21.8% of RCC patients admitted in emergency mode [30]. Similarly, our study observed a higher rate of emergency admissions in LCC (13.27%) compared to RCC (5.59%), but the difference was not statistically significant (*p* = 0.07). This trend could be linked to the higher incidence of colon obstruction in LCC, as noted by Bourakkadi et al., where obstruction was present in 45.5% of LCC and 31.3% of RCC cases [31]. However, in our study, colon obstruction rates were comparable between RCC and LCC (8.7% vs. 10.31%, *p* = 0.71), which may have mitigated differences in emergency admission rates.

Early postoperative complications occurred in 31 patients (16.40%), with similar rates between RCC (17.58%) and LCC (15.31%) groups (*p* = 0.72). These findings align with reported postoperative morbidity rates in the literature, which range from 10.8% to 19.2% for RCC and 5.7% to 19.78% for LCC [29,31]. In our study, emergency admission was identified as an independent positive predictive factor for postoperative complications (*p* < 0.001, OR = 6.24, 95% CI = 2.18–17.86). This aligns with findings by Havens et al. who demonstrated a higher risk of complications with emergency admissions (OR = 1.39, 95% CI = 1.03–1.86) [32]. Sørensen et al. also reported that emergency abdominal surgery doubles the complication rate compared to elective procedures [33]. This may be explained by the poorer preoperative clinical condition of patients requiring emergency surgery, often characterized by dyselectrolytemia, peritonitis, dehydration, and severe inflammation—factors consistently linked to increased postoperative risks [34,35,36].

Arguably, one of the most significant differences between RCC and LCC is the tendency for more advanced cancer stages at the time of diagnosis among patients with RCC compared to those with LCC. Studies report that 26.8–49.2% of RCC patients are diagnosed with AJCC stage III colon cancer, compared to 23.5–37% of LCC patients. Stage IV cancer was found in 3.7–27% of RCC patients and 3.5–17.8% of LCC patients [22,30,37]. However, in our study, no significant difference was observed in AJCC stages: 24 (26.37%) RCC patients and 21 (20.41%) LCC patients were diagnosed with stage III, while 5 (5.49%) RCC and 13 (13.27%) LCC patients had stage IV (*p* = 0.25). This may be due to earlier diagnosis in LCC patients, whose symptoms (e.g., hematochezia) tend to be more alarming, leading to earlier medical attention. Conversely, RCC symptoms (e.g., abdominal pain and anemia) are often nonspecific, contributing to later detection. Disparities between our study and existing literature may be linked to the COVID-19 pandemic, which delayed cancer diagnoses and disrupted stage distributions between RCC and LCC.

Another notable difference between RCC and LCC is the histological type of the tumor. Research indicates that patients with RCC are more frequently diagnosed with mucinous tumors compared to patients with LCC. The occurrence of mucinous tumors in RCC ranges from 4% to 15.5%, whereas in LCC it ranges from 1% to 5.8% [6,28,37,38]. In our group, we have noticed a similar pattern. Mucinous tumors in our patients were diagnosed in nine (9.89%) patients with RCC and in one (1.02%) patient with LCC; *p* = 0.008. Unfortunately, the reason for this difference remains unknown [38].

We observed that patients with RCC had a greater number of lymph nodes isolated compared to those with LCC (17 vs. 13; *p* = 0.002). This finding aligns with previous studies, where similar trends have been reported by other researchers, suggesting that patients with RCC generally have more lymph nodes retrieved during surgical procedures than those with LCC [16,22]. For example, Lee et al. found that the average number of lymph nodes harvested in patients with RCC was 19.2, whereas for those with LCC it was 16.5 [16]. This discrepancy can be attributed to the fact that, in our study as well as in the literature, the most common tumor localization in LCC is the sigmoid colon, which typically results in the performance of a sigmoidectomy. Consequently, patients with LCC tend to have fewer lymph nodes harvested during the procedure compared to those with RCC. This is due to the fact that a right hemicolectomy, which is the most frequently performed in patients with RCC, is a more extensive procedure than a sigmoidectomy, involving a larger segment of the colon and a more comprehensive removal of the associated lymphatic tissue.

In the case of colon cancer, significant attention is directed towards identifying factors that may impact postoperative survival. Several studies propose that independent predictive factors for OS include cancer localization (RCC or LCC), AJCC staging, and the number of isolated lymph nodes. A study conducted by Hadges et al. demonstrated that LCC was a predictive factor for OS (*p* = 0.003; HR = 0.845, 95% CI = 0.756–0.944) [F]. Conversely, Yang et al. found no significant difference between RCC and LCC in OS (*p* = 0.11; HR = 1.23, 95% CI = 0.96–1.57) [30]. In our study, we observed similar results to those of Yang et al., where cancer localization had no significant impact on OS among our patients (*p* = 0.83; HR = 1.13, 95% CI = 0.42–3.06). The differences between studies in terms of the impact of localization on OS might be associated with the phenomenon mentioned earlier: RCC tumors tend to have higher AJCC staging than LCC tumors, potentially resulting in worse survival outcomes. Both our study and the study by Yang et al. did not observe any differences between tumor localization and AJCC staging. Therefore, in both studies, the localization of colon cancer had no impact on OS.

Another predictive factor for OS in patients with colon cancer is AJCC staging. Studies conducted by Mangone et al. and Bustamante-Lopez et al. have shown that stages III and IV significantly decrease the OS in patients with colon cancer (*p* < 0.001, HR = 2.01, 95% CI = 1.65–2.44; *p* < 0.001, HR = 3.23, 95% CI = 2.01–5.17, respectively) [22,37]. We found a similar result that stage III–IV can be an independent predictive factor for OS (*p* = 0.02; HR = 6.33, 95% CI = 1.28–31.32). This can be associated with the fact that AJCC stage III and IV include patients with lymph nodes or distant metastasis. Therefore, metastatic colon cancer significantly decreases patients’ survival. Additionally, the difference in median OS between the stage I–II group and the stage III–IV group can be associated with the treatment year. In the group of patients with stage III–IV, 22.58% of deaths were observed, compared to 1.57% in the stage I–II group. The higher median survival in the stage III–IV group (11.5 (0.5–60, IQR 26) vs. 10 (0.5–63.5, IQR 21) months in I–II group) is most likely due to the fact that the majority of these patients underwent surgical treatment between 2018 and 2022. Despite the higher mortality rate, this longer follow-up period results in a greater median OS. In contrast, the stage I–II group includes a significant number of patients who were treated in 2023, which shortens the observed survival time due to a shorter follow-up period.

In our study, no difference in OS was observed between patients with more than 21, 12–21, and fewer than 12 isolated lymph nodes (*p* = 0.52; HR = 1.33, 95% CI = 0.38–4.61 and *p* = 0.62; HR = 0.89, 95% CI = 0.27–2.94). However, recent studies highlight this difference. Mangone et al., found better OS for patients with 12–21 (HR = 0.53, 95% CI = 0.40–0.72) and >21 isolated lymph nodes (HR = 0.40, 95% CI = 0.30–0.55) regardless of AJCC stage [22], a finding confirmed by Lee et al. (HR = 0.85, 95% CI = 0.83–0.87 for 12–21 and HR = 0.82, 95% CI = 0.80–0.84 for >21 lymph nodes) [16]. The reason for this pattern is unclear, and further studies are needed to determine whether the number of removed lymph nodes reflects a biological characteristic influencing prognosis or the therapeutic impact of lymph node removal itself.

This study has several limitations. First of all, the retrospective character of this study, conducted in a single medical center. In addition, some years of this study overlapped with the COVID-19 pandemic, which could delay diagnosis of patients between 2020 and 2022, which could result in more advanced tumor staging.

## 5. Conclusions

Our study confirms the trend of increasing incidence of colorectal cancer, consistent with data from the Polish National Cancer Registry and observed Western trends in countries such as Canada, Sweden, and the United Kingdom. Based on our findings, the national screening program likely plays a key role in the early detection of colon tumors, as evidenced by the higher number of asymptomatic cases identified through screening colonoscopy. What is more, the highest increase in incidence of colorectal cancer was observed in the post pandemic period, which was associated with limited access to diagnostic and treatment methods during the COVID-19 pandemic. Additionally, patients with RCC tend to be older compared to patients with LCC. The frequency of postoperative complications does not differ between patients with RCC and LCC. However, the emergency hospital admission mode proved to be a significant predictive factor for postoperative complications. Furthermore, patients with RCC are more frequently diagnosed with mucinous tumors compared to patients with LCC. Finally, there is no difference in overall survival between patients with RCC and LCC. Nonetheless, the AJCC stage and scope of the surgery emerged as significant predictive factors for overall survival.

## Figures and Tables

**Table 1:** Patients’ general characteristics.

Cancer Localization	RCC (91/189; 48.15%)	LCC (98/189; 51.85%)	Total (189)	*p*
Age (years)	70 (36–92, IQR 11)	68 (38–84, IQR 12.5)	69 (36–92, IQR 11)	0.02
Gender				
Male	52 (56.52%)	51 (52.58%)	103 (54.50%)	0.59
Female	40 (43.48%)	46 (47.42%)	86 (45.50%)	
BMI	25.63 (17.47–47.11, IQR 5.10)	26.85 (17.63–41.66, IQR 5.72)	26.27 (17.47–47.11, IQR 5.24	0.67
Current cigarette smoking (yes)	16 (17.39%)	16 (16.49%)	32 (16.39%)	0.86
Presence of comorbidities (arterial hypertension, diabetes mellitus, history of myocardial infarction/stroke, COPD, asthma, and others)	70 (73.68%)	69 (71.13%)	139 (73.54%)	0.69
Ulcerative colitis (yes)	4 (4.40%)	1 (1.03%)	5 (2.60%)	0.21
Family history of colon cancer (yes)	8 (8.70%)	13 (13.40%)	21 (11.11%)	0.30
Clinical symptoms (yes)	54 (58.7%)	53 (54.64%)	107 (56.61%)	0.57
Abdominal pain	30 (32.61%)	30 (30.93%)	60 (31.75%)	0.81
Weight loss	19 (20.65%)	22 (22.68%)	41 (21.69%)	0.73
Hematochezia	10 (10.87%)	18 (18.56%)	28 (14.81%)	0.13
Anemia	20 (21.74%)	6 (6.19%)	26 (13.76%)	0.002
Nausea and vomiting	10 (10.87%)	10 (10.30%)	20 (10.62%)	0.93
Ileus	8 (8.70%)	10 (10.31%)	18 (9.52%)	0.71
Body weakness	10 (10.87%)	5 (5.21%)	15 (7.98%)	0.15
Diarrhea	6 (6.52%)	7 (7.22%)	13 (6.88%)	0.87
Constipation	6 (6.52%)	7 (7.22%)	13 (6.88%)	0.87
Melaena	2 (2.17%)	2 (2.06%)	4 (2.12%)	1
Colon perforation	1 (1.10%)	0 (0%)	1 (0.53%)	0.48
Albumin (g/dL)	4.30 (2.20–5.10), IQR 0.52	4.19 (2.86–5.08), IQR 0.86	4.30 (2.20–5.10), IQR 0.65	0.91
Hemoglobin (g/dL)	11.80 (6.20–18), IQR 4.6	12.50 (8–15.80), IQR 3.60	12.40 (6.20–18), IQR 3.90	0.65
NRS 2002	2 (2–6), IQR 1	2 (2–5), IQR 1	2 (2–6), IQR 1	0.71

Abbreviations: RCC—Right colon cancer, LCC—Left colon cancer, IQR—interquartile range, BMI—body mass index, COPD—Chronic obstructive pulmonary disease, NRS 2002—nutrition risk score 2002.

**Table 2:** Surgical procedures characteristics.

Cancer Localization	RCC (91/189; 48.15%)	LCC (98/189; 51.85%)	Total (189)	*p*
Admission mode				
Elective admission	86 (94.51%)	85 (86.73%)	171 (90.48%)	0.07
Emergency admission	5 (5.59%)	13 (13.27%)	18 (9.52%)	
ASA score				
II	43 (47.25%)	46 (46.94%)	89 (47.09%)	0.97
III	44 (48.35%)	47 (47.96%)	91 (48.15%)	
IV	4 (4.40%)	5 (5.10%)	9 (4.76%)	
Scope of surgery				
Radical tumor resection	88 (96.70%)	88 (89.80%)	176 (93.12%)	0.08
Palliative resection	3 (3.30%)	10 (10.20%)	13 (6.88%)	
Type of surgical access				
Laparotomy	49 (53.85%)	54 (55.10%)	103 (54.50%)	0.86
Laparoscopy	42 (46.15%)	44 (44.90%)	86 (45.50%)	
Type of the surgery				
Right hemicolectomy	71 (78.02%)	0 (0%)	71 (37.57%)	
Sigmoidectomy	0 (0%)	69 (70.41%)	69 (36.51%)	
Left hemicolectomy	0 (0%)	18 (18.37%)	18 (9.52%)	
Extended right hemicolectomy	15 (16.48%)	0 (0%)	15 (7.94%)	
Extended left hemicolectomy	0 (0%)	6 (6.12%)	6 (3.17%)	
Transverse colon resection	1 (1.10%)	5 (5.10%)	6 (3.17%)	
Total colectomy	4 (4.40%)	0 (0%)	4 (2.12%)	
Resection margin				
R0	91 (100%)	96 (97.96%)	187 (98.94%)	0.49
R1	0 (0%)	0 (0%)	0 (0%)	
R2	0 (0%)	2 (2.04%)	2 (1.06%)	
Primary anastomosis (yes)	79 (86.81%)	74 (75.51%)	153 (80.95%)	0.05
Type of anastomosis				
Side-to-side ileo-transversostomy	60 (65.94%)	0 (0%)	60 (31.75%)	
End-to-end descendo-rectostomy	0 (0%)	45 (48.98%)	45 (23.81%)	
End-to-end transverso-sigmoidostomy	0 (0%)	22 (22.45%)	22 (11.64%)	
Side-to-end ileo-transversostomy	14 (15.38%)	0 (0%)	14 (7.41%)	
End-to-end descendo-sigmoidostomy	0 (0%)	4 (4.08%)	4 (2.12%)	
End-to-side ileo-transversostomy	4 (4.40%)	0 (0%)	4 (2.12%)	
End-to-end sigmoido-sigmoidostomy	0 (0%)	2 (2.04%)	2 (1.06%)	
Side-to-side ascendo-descendostomy	0 (0%)	1 (1.02%)	1 (0.53%)	
Side-to-side ileo-sigmoidostomy	1 (1.10%)	0 (0%)	1 (0.53%)	
Stoma (yes)	12 (13.19%)	24 (24.49%)	36 (19.05%)	0.05
Type of the stoma (time type)				
Definitive stoma	12 (13.19%)	18 (18.36%)	30 (15.87%)	0.04
Reversal stoma	0 (0%)	6 (6.12%)	6 (3.17%)	
Type of the stoma (construction type)				
End stoma	12 (13.19%)	20 (20.41%)	32 (16.93%)	0.04
Loop stoma	0 (0%)	4 (4.08%)	4 (2.12%)	
Stoma Localization				
Descending colostomy	0 (0%)	19 (19.39%)	19 (10.05%)	
Ileostomy	10 (10.98%)	0 (0%)	10 (5.29%)	
Transverse colostomy	0 (0%)	5 (5.10%)	5 (2.65%)	
Ascending colostomy	2 (2.20%)	0 (0%)	2 (1.06%)	
Lymph nodes isolated	17 (1–45, IQR 12)	13 (1–57, IQR 10)	14 (1–57, IQR 10)	0.002
Treatment of liver metastasis (yes)	2 (2.20%)	3 (3.06%)	5 (2.65%)	1
Type of the surgical treatment of liver metastasis				
Anatomical liver resection	1 (1.10%)	3 (3.06%)	4 (2.12%)	
Non-anatomical liver resection	1 (1.10%)	0 (0%)	1 (0.53%)	
Blood loss				
<400 mL	88 (96.70%)	95 (96.94%)	183 (96.83%)	1
>400 mL	3 (3.30%)	3 (3.06%)	6 (3.17%)	
Duration of the procedure (minutes)	225 (120–420, IQR 80)	210 (105–505, IQR 85)	215 (105–505, IQR 85)	0.16
Complications	16 (17.58%)	15 (15.31%)	31 (16.40%)	0.72
Wound infection	5 (5.49%)	1 (1.02%)	6 (3.17%)	0.11
Wound dehiscence	1 (1.10%)	5 (5.10%)	5 (2.65%)	0.37
Colon obstruction	2 (2.20%)	2 (2.04%)	4 (2.12%)	1
Descending colostomy obstruction	0 (0%)	2 (2.04%)	2 (1.06%)	
Ileo-transversostomy obstruction	1 (1.10%)	0 (0%)	1 (0.53%)	
Ileostomy obstruction	1 (1.10%)	0 (0%)	1 (0.53%)	
Intestine perforation	0 (0%)	3 (3.06%)	3 (1.59%)	0.11
Descending colon perforation	0 (0%)	2 (2.04%)	2 (1.06%)	
Ileum perforation	0 (0%)	1 (1.02%)	1 (0.53%)	
Anastomotic leakage	3 (3.30%)	0 (0%)	3 (1.59%)	0.11
Ileo-transverse anastomosis leakage	3 (3.30%)	0 (0%)	3 (1.59%)	
Sepsis	2 (2.20%)	0 (0%)	2 (1.06%)	0.24
Intra-abdominal hemorrhage	1 (1.10%)	0 (0%)	1 (0.53%)	0.48
Intra-abdominal abscess	0 (0%)	1 (1.02%)	1 (0.53%)	1
Cutaneo-vesical fistula	0 (0%)	1 (1.02%)	1 (0.53%)	1
Clostridium difficile infection	1 (1.10%)	0 (0%)	1 (0.53%)	0.48
Colo-cutaneus fistula	1 (1.10%)	0 (0%)	1 (0.53%)	0.48
Iatrogenic ureteral perforation	0 (0%)	1 (1.02%)	1 (0.53%)	1
Acute ischemia of ileum	0 (0%)	1 (1.02%)	1 (0.53%)	1
Reoperations	4 (4.40%)	6 (6.12%)	10 (5.29%)	0.49
30 day mortality	2 (2.20%)	2 (2.04%)	4 (2.12%)	1
Duration of hospitalization (days)	8 (1–36, IQR 4)	8 (1–32, IQR 4)	8 (1–36, IQR 4)	0.92
Rehospitalization (yes)	3 (3.30%)	4 (4.08%)	7 (3.70%)	1

Patients had more than one postoperative complication. Abbreviations: RCC—Right colon cancer, LCC—Left colon cancer, ASA—American Society of Anesthesiologists, IQR—interquartile range.

**Table 3:** Univariate and multivariate logistic regression analysis for predictive factors of postoperative complications.

	Univariate Analysis				Multivariate Analysis		
	*n*	OR	95% CI	*p* (df = 1)	OR	95% CI	*p* (df = 1)
Age		1.02	0.95–1.08	0.49			
Gender							
Male	103	0.28	0.41–4.33	0.63			
Female	86	1					
BMI		1.03	0.99–1.07	0.15			
Presence of comorbidities (yes)	139	1.55	0.43–5.55	0.49			
Cigarette smoking (yes)	32	2.62	0.66–10.38	0.18			
Clinical symptoms (yes)	107	0.83	0.26–2.70	0.76			
ASA score							
≥III	100	2.1	0.92–4.77	0.07			
≤II	89	1					
Tumor origin							
LCC	98	1.15	0.53–2.51	0.72			
RCC	91	1					
Admission mode							
Emergency mode	18	6.77	2.41–19.03	<0.001	6.24	2.18–17.86	<0.001
Elective mode	171	1			1		
Surgical approach							
Laparotomy	103	1		0.22			
Laparoscopy	86	0.61	0.27–1.36				
Intent of surgery							
Radical tumor resection	176	1		0.046			0.1
Palliative resection	13	3.61	1.09–11.97		2.97	0.82–10.75	
Blood loss							
<400 mL	183	1		0.30			
>400 mL	6	2.66	0.46–15.35				
Duration of surgical procedure	1	0.99–1	0.26	0.26			

Abbreviations: BMI—Body mass index, OR—odds ratio, CI—confidence interval, RCC—Right colon cancer, LCC—Left colon cancer, ASA—American Society of Anesthesiologists.

**Table 4:** Indications and type of the reoperations in patients after primary treatment for RCC and LCC.

Cancer Localization	Indication for Reoperation	Type of Reoperations
RCC	Ileo-transverse anastomosis leakage (2; 2.20%)	Loop ileostomy (1; 1.10%)
		Ileo-transverse anastomosis repair (1; 1.10%)
	Colon obstruction (1; 1.10%)	Ileostomy (1; 1.10%)
	Intra-abdominal hemorrhage	Exploratory laparotomy (1; 1.10%)
LCC	Intestine perforation (3; 3.06%)	End ileostomy (2; 2.04%)
		Loop ileostomy (1; 1.02%)
	Wound dehiscence (2; 2.04%)	Wound dehiscence revision surgery (2; 2.04%)
	Descending colostomy obstruction (1; 1.02%)	Hartmann’s procedure

Abbreviations: RCC—Right colon cancer, LCC—Left colon cancer.

**Table 5:** Histopathological tumor data.

Cancer Localization	RCC (91/189; 48.15%)	LCC (98/189; 51.85%)	Total (189)	*p*
Tumor localization				
Sigmoid colon	0 (0%)	75 (76.53%)	75 (16.38%)	
Ascending colon	32 (35.16%)	0 (0%)	32 (6.99%)	
Hepatic flexure	23 (25.27%)	0 (0%)	23 (5.02%)	
Caecum	20 (21.98%)	0 (0%)	20 (4.36%)	
Proximal ⅔ of transverse colon	15 (16.48%)	0 (0%)	15 (3.28%)	
Descending colon	0 (0%)	9 (9.18%)	9 (1.97%)	
Splenic flexure	0 (0%)	8 (8.16%)	8 (1.75%)	
Distal ⅓ of transverse colon	0 (0%)	6 (6.12%)	6 (1.31%)	
Ileocecal valve	1 (1.09%)	0 (0%)	1 (0.53%)	
Tumor size (mm)	40 (2–220, IQR 38)	35 (4–450, IQR 23)	38 (2–450, IQR 27)	0.41
Histological type				
Adenocarcinoma	82 (90.11%)	97 (98.98%)	179 (94.71%)	0.08
Mucinous carcinoma	9 (9.89%)	1 (1.02%)	10 (5.29%)	
Grading				
G1	13 (14.29%)	22 (22.45%)	35 (18.52%)	0.08
G2	70 (75.92%)	73 (74.49%)	143 (75.66%)	
G3	8 (8.79%)	3 (3.06%)	11 (5.82%)	
Pathological staging				
T				
0	2 (2.20%)	1 (1.02%)	3 (1.59%)	0.95
1	15 (16.48%)	14 (14.29%)	29 (15.34%)	
2	22 (24.18%)	24 (24.49%)	46 (24.34%)	
3	38 (41.76%)	43 (43.88%)	81 (42.86%)	
4	14 (15.38%)	16 (16.33%)	30 (15.87%)	
N				
0	62 (68.13%)	68 (69.39%)	13 (68.78%)	0.48
1	17 (18.68%)	22 (22.45%)	39 (20.63%)	
2	12 (13.19%)	8 (8.16%)	20 (10.58%)	
M				
0	85 (93.41%)	85 (86.73%)	170 (89.95%)	0.07
1	5 (5.49%)	13 (13.27%)	18 (9.52%)	
AJCC Stage				
0	2 (2.20%)	1 (1.02%)	3 (1.59%)	0.25
I	33 (36.26%)	33 (33.67%)	66 (34.92%)	
II	27 (29.67%)	31 (31.63%)	58 (30.69%)	
III	24 (26.37%)	21 (20.41%)	45 (23.81%)	
IV	5 (5.49%)	13 (13.27%)	18 (9.52%)	
Localization of distant metastasis *				
Liver	5 (5.49%)	10 (10.20%)	15 (7.94%)	0.23
Lungs	1 (1.10%)	0 (0%)	1 (0.53%)	0.48
Peritoneum	0 (0%)	1 (1.02%)	1 (0.53%)	1
Lymphovascular invasion (yes)	22 (24.18%)	20 (20.41%)	42 (22.22%)	0.53
Perineural invasion (yes)	6 (6.59%)	5 (5.10%)	11 (5.82%)	0.66

* One patient with RCC had lung and liver metastasis simultaneously. Abbreviations: RCC—Right colon cancer, LCC—Left colon cancer, IQR—interquartile range.

**Table 6:** Follow-up.

Cancer Localization	RCC (91/189; 48.15%)	LCC (98/189; 51.85%)	Total (189)	*p*
Follow-up duration (months)	11 (0.5–63.50, IQR 24.50)	10.75 (0.5–61.50, IQR 22.00)	11 (0.5–63.50, IQR 23.50)	0.60
Chemotherapy (yes)	12 (13.19%)	31 (31.63%)	43 (22.75%)	0.003
FOLFOX4	2 (2.20%)	11 (11.22%)	13 (6.88%)	0.02
FOLFIRI	3 (3.30%)	10 (10.20%)	13 (6.88%)	0.08
XELOX	2 (2.20%)	5 (5.10%)	7 (3.70%)	0.44
LF4	2 (2.20%)	2 (2.04%)	4 (2.12%)	1
mFOLFOX4	1 (1.10%)	1 (1.02%)	2 (1.06%)	1
LF1	0 (0%)	1 (1.02%)	1 (0.53%)	1
Carboplatin	1 (1.10%)	0 (0%)	1 (0.53%)	0.48
mFOLFOX6	0 (0%)	1 (1.02%)	1 (0.53%)	1
De Gramont	1 (1.10%)	0 (0%)	1 (0.53%)	0.48
Radiotherapy (yes)	0 (0%)	3 (3.06%)	3 (1.59%)	0.25
Recurrence (yes)	0 (0%)	2 (2.04%)	2 (1.06%)	0.49
One-year overall survival	91.57% (SE 3.43%)	93.99% (SE 2.61%)	92.76% (SE 2.16%)	0.79
Estimated 5-year overall survival	87.58% (SE 5.10%)	76.33 (SE 8.79%)	85.25% (SE 4.11%)	0.79

Abbreviations: RCC—Right colon cancer, LCC—Left colon cancer, IQR—interquartile range, SE—standard error.

**Table 7:** Uni- and multivariate analysis of predictive factors for overall survival in the whole cohort.

	Univariate Analysis				Multivariate Analysis		
Variate	Survival Time (Months)	HR	95% CI	*p* (df = 1)	HR	95% CI	*p* (df = 1)
Age		0.99	0.95–1.04	0.77			
Gender							
Male	9.75 (0.5–63.50, IQR 20)	0.89	0.33–2.39	0.54			
Female	13.50 (0.5–61.50, IQR 26.50	1					
Tumor origin							
LCC	10.75 (0.5–61.50, IQR 22.00)	1.13	0.42–3.06	0.79			
RCC	11 (0.5–63.50, IQR 24.50)	1					
AJCC Stage							
0-II	10 (0.5–63.5, IQR 21)			<0.001	1		0.02
III-IV	11.5 (0.5–60, IQR 26)	13.37	3.04–58.89		6.33	1.28–31.32	
Lymph nodes isolated							
<12	10.25 (0.5–60.5, IQR 19.5)	1					
12–21	11.75 (0.5–63.5, IQR 26.25)	0.89	0.27–2.94	0.62			
>21	10 (1–50, IQR 24)	1.33	0.38–4.61	0.53			
Intent of surgery							
Radical tumor resection	11 (0.5–63.5, IQR 25)	1		<0.001	1		<0.001
Palliative resection	9.5 (2.5–45, IQR 21.5)	18.44	6.85–49.66		8.27	2.84–24.05	
Postoperative chemotherapy							
Yes	18 (2.5–60, IQR 21)	2.52	0.95–6.78	0.06			
No	9.25 (0.5–63.5, IQR 21	1					
Tumor diameter	1	0.99–1.01	0.91	0.91			0.91
Lymphovascular invasion							
Yes	8.25 (0.5–52.5, IQR 20	1.31	0.42–4.07	0.64			
No	11 (0.5–63.5, IQR 24)	1					

Abbreviations: HR—hazard ratio, CI—confidence interval, RCC—Right colon cancer, LCC—Left colon cancer, IQR—interquartile range.

## Data Availability

The original contributions presented in this study are included in the article. Further inquiries can be directed to the corresponding author(s).
